# Pancreatic cancer cell-derived IGFBP-3 contributes to muscle wasting

**DOI:** 10.1186/s13046-016-0317-z

**Published:** 2016-03-15

**Authors:** Xiu-yan Huang, Zi-li Huang, Ju-hong Yang, Yong-hua Xu, Jiu-Song Sun, Qi Zheng, Chunyao Wei, Wei Song, Zhou Yuan

**Affiliations:** Department of General Surgery, Shanghai Jiaotong University Affiliated Sixth People’s Hospital, Shanghai, 200233 P.R. China; Department of Radiology, Xuhui Central Hospital, Shanghai, 200031 PR China; Collaborative Innovation Center of Tianjin for Medical Epigenetics, Key Laboratory of Hormone and Development (Ministry of Health), Metabolic Disease Hospital & Tianjin Institute of Endocrinology, Tianjin Medical University, Tianjin, 300070 China; Department of Cancer Immunology and AIDS, Dana-Farber Cancer Institute, 450 Brookline Ave., Boston, MA 02215 USA; Department of Medicine, Harvard Medical School, 25 Shattuck Street, Boston, MA 02115 USA; Howard Hughes Medical Institute; Department of Molecular Biology, Massachusetts General Hospital, Boston, MA USA; Department of Genetics, Harvard Medical School, Boston, MA 02115 USA

## Abstract

**Background:**

Progressive loss of skeletal muscle, termed muscle wasting, is a hallmark of cancer cachexia and contributes to weakness, reduced quality of life, as well as poor response to therapy. Previous studies have indicated that systemic host inflammatory response regarding tumor development results in muscle wasting. However, how tumor directly regulates muscle wasting via tumor-derived secreted proteins is still largely unknown.

**Methods:**

In this study, we performed bioinformatics analysis in two datasets of pancreatic ductal adenocarcinoma, which causes cancer cachexia and muscle wasting with the highest prevalence, and uncovered that *IGFBP3*, which encodes IGF-binding protein-3 (IGFBP-3), is dramatically up-regulated in pancreatic tumor samples. We also verified the wasting effect of IGFBP-3 on C2C12 muscle cells with biochemical and genetic assays.

**Results:**

IGFBP-3 potently leads to impaired myogenesis and enhanced muscle protein degradation, the major features of muscle wasting, via IGF signaling inhibition. Moreover, conditioned medium from Capan-1 pancreatic cancer cells, which contains abundant IGFBP-3, significantly induces muscle cell wasting. This wasting effect is potently alleviated by *IGFBP3* knockdown in Capan-1 cells or IGFBP-3 antibody neutralization. Strikingly, compared to muscle cells, IGF signaling and proliferation rate of Capan-1 cells were rarely affected by IGFBP-3 treatment.

**Conclusions:**

Our results demonstrated that pancreatic cancer cells induce muscle wasting via IGFBP-3 production.

**Electronic supplementary material:**

The online version of this article (doi:10.1186/s13046-016-0317-z) contains supplementary material, which is available to authorized users.

## Background

Patients with pancreatic cancer often develop the most severe degrees of cachexia that is highly associated with cancer death [1]. Clinically, cancer cachexia is defined as an unintentional 10 % loss of body weight over 12 months [2]. Previous studies have indicated that the progressive loss of skeletal muscle, termed muscle wasting, is a key phenotype of cancer cachexia and results in weakness, reduced ambulation, diminished quality of life, poor response to therapy, as well as death due to respiratory failure or infection [3]. However, approved effective treatments for muscle wasting in pancreatic cancer patients are still missing. Thus, understanding the molecular mechanisms of muscle wasting will provide novel insight into developing targeted therapies and improving the quality of life for pancreatic cancer patients and, possibly, for other malignancies.

There are increasing evidences that both impaired myogenesis and increased muscle protein degradation contribute to muscle wasting during cancer cachexia [4–6]. Systemic hormones have been shown to regulate these biological processes. For example, TGFβ superfamily members, including activin A, GDF15, as well as Myostatin, can cause muscle loss through SMAD signaling [4, 7, 8]. Systemic inflammatory cytokines, including TNFα, IL-1α, IL-1β, IL-6 and related ligands haven been shown to cause muscle wasting in both mouse models and human samples [9]. Growing studies across different species indicated that tumor-derived hormones also play essential roles for muscle wasting. For example, conditioned medium from pancreatic cancer cells that contains numerous cancer-derived peptides, including Myostatin and activin A, is sufficient to cause muscle wasting [4, 10, 11]. In addition, tumor-derived parathyroid-hormone-related protein (PTHrP) has been shown to induce muscle wasting and lipid depletion in a mouse model [12]. An insulin-like binding protein, ImpL2, is secreted from tumor-like cells and impairs muscle function and systemic tissue growth via inhibition of IGF-like signaling in *Drosophila* [13–15]. Thus, revealing how tumor-derived secreted proteins cause muscle wasting will shed the light on novel mechanisms of tumor-host interaction regarding cancer cachexia.

Mammalian insulin-like growth factor binding protein (IGFBP) 1–7 and *Drosophila* ImpL2 share high homology in structures or functions. Classically, IGFBPs bind to insulin-like growth factors (IGFs) to stabilize the complex and enhance the half-life and distribution of IGFs to target tissues. On the other hand, excess IGFBPs restrain the bio-ability of IGFs to their receptors and suppress intracellular IGF signaling that is required for myogenesis and myotube atrophy [16–18]. The notion is further supported by the evidence that endogenous IGFBP-5 has been shown to promote myogenesis via activation of IGF-2/AKT/FoxO signaling, whereas, IGFBP-5 overexpression tremendously causes retarded muscle development [19, 20]. In addition to IGF signaling, IGFBPs also regulate cell biological processes via other signaling pathways, including NF-κB, TGF-β, JAK/STAT, and heat shock protein signalings [21, 22]. Notably, injection of IGF-1/IGFBP-3 complex improves weight loss in tumor-bearing mice [23]. However, whether excess IGFBPs are secreted from tumors to regulate muscle wasting is far less established. In this study we analyzed the gene expression profile and identified that *IGFBP3* is dramatically induced in pancreatic tumors. We further demonstrated that IGFBP-3, which is abundantly produced in pancreatic cancer cells, causes muscle wasting through both impaired myogenesis and enhanced myotube protein degradation via, at least, inhibition of IGF/PI3K/AKT signaling. Thus we propose that pancreatic tumors result in muscle wasting via secretion of IGFBP-3.

## Results

### Secreted protein genes are induced in pancreatic tumors

**Fig. 1:**
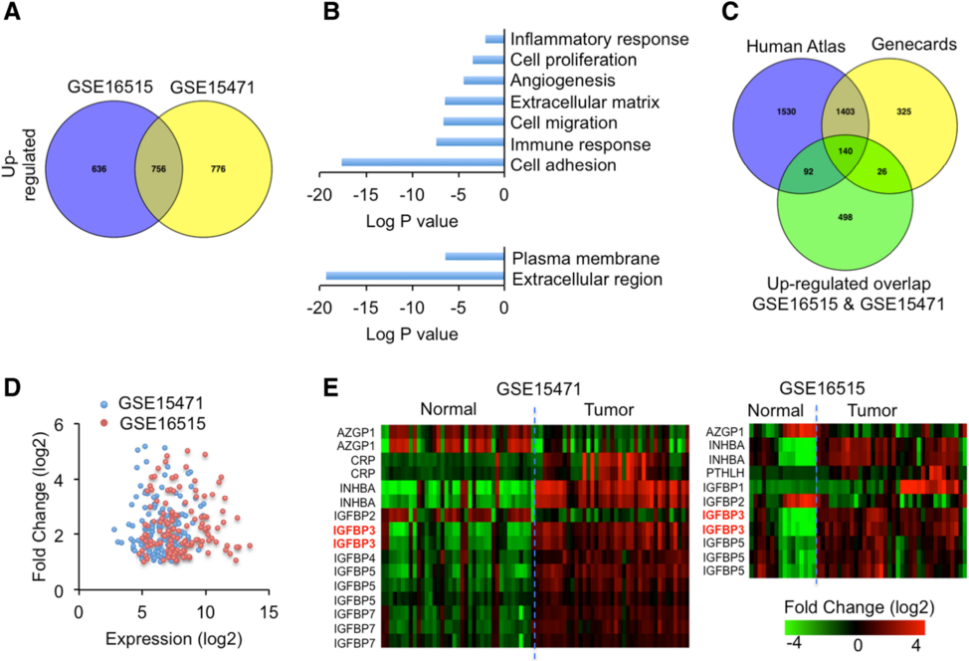
Secreted protein genes are induced in pancreatic tumors.**a** Significantly up-regulated genes (fold change > = 2, FDR < 0.05) were identified in two pancreatic tumor microarray datasets. The number of overlapping genes in both was indicated in the middle. **b** Significantly enriched (EASE score < 0.05) GO terms were identified in overlapping up-regulated gene list (Up, biological process. Down, cellular component). **c** Venn diagram indicated the overlap in Human Atlas secreted-protein-gene database, GeneCards secreted-protein-gene database, and up-regulated gene list in both pancreatic tumor datasets. **d** Overlapping up-regulated genes in both datasets were indicated with basal expression level (probe signal) and fold change. **e** The differential expression patterns of cachexia-related and IGFBP family genes were indicated in heatmaps. The intensity reflected fold change

In order to study whether secreted proteins that likely regulate tumor-host crosstalk are up-regulated in pancreatic tumors, we analyzed the gene expression profile in both pancreatic tumors and normal pancreatic tissues. Here two different datasets (GSE15471, 36 normal-tumor sample pairs, and GSE16515, 36 tumor samples and 16 normal samples) were used for bioinformatics analysis. Subsequently, we found that 756 genes were up-regulated and 160 genes were down-regulated in both pancreatic tumor samples compared to normal tissues (fold change > = 2 and FDR < 0.05) (Fig. 1a, Additional file 1: Figure S1A-S1C, Additional file 2: Table S1 & Additional file 3: Table S2). Gene ontology (GO) enrichment analysis of signaling pathways revealed that overlapping up-regulated genes involved in TGF-β, integrin, PDGF, as well as p53 signalings, are significantly enriched (Additional file 1: Figure S1D). Further, significant enrichment of multiple tumorigenic biological processes, including inflammatory response, cell proliferation, angiogenesis, extracellular matrix, cell migration, immune response, and cell adhesion, was observed in overlapping up-regulated genes (Fig. 1b, up). Interestingly, GO enrichment analysis regarding cellular component also revealed significant enrichment of “plasma membrane” and “extracellular region” where secreted protein are processed and secreted (Fig. 1b, down). However, GO enrichment analysis of overlapping down-regulated genes uncovered that only “amino acid metabolism” biological process was significantly enriched (Additional file 1: Figure S1E).

To test which secreted proteins are induced in tumor samples, we applied 756 up-regulated genes into “Human Atlas” (http://www.proteinatlas.org/) and “Genecards” (http://www.genecards.org/) gene-annotation databases. 232 genes (30.7 %) that encode secreted proteins were found in “Human Atlas” database, 166 (22 %) in “Genecards” database, and 140 (18.5 %) in both (Fig. 1c-d and Additional file 3: Table S2).

Previous studies have indicated that a few cytokines or secreted proteins are associated with cancer cachexia. As patients with pancreatic cancer are under great prevalence to develop cancer cachexia and muscle wasting, we hypothesized that the expression of secreted proteins associated to muscle wasting is predominantly induced in pancreatic tumors. We measured the fold changes of cancer cachexia-associated secreted protein genes in pancreatic tumor samples. These secreted protein genes include systemic inflammatory factors, *CRP* (encodes C-reactive protein) [24], *IL1A* (encodes interleukin-1α) [9], *IL1B* (encodes interleukin-1β) [9], *IL6* (encodes interleukin-6) [9], *IL10* (encodes interleukin-10) [9], *TNF* (encodes Tumor necrosis factors α) [9]; TGF-β families, *GDF15* (encodes growth differentiation factor 15) [25], *MSTN* (encodes myostatin) [4], *INHBA* (encodes inhibin βA subunit of Activin A and AB) [26], *INHBB* (encodes inhibin βB) [27]; others, *AZGP1* (encodes Zinc-α2-glycoprotein) [28], *DCD* (encodes human cachexia-associated protein) [29], as well as *PTHLH* (encodes parathyroid hormone-like hormone) [12]. However, among above secreted protein genes, only *INHBA* is dramatically increased in both pancreatic tumor datasets compared to normal tissues. *PTHLH* is up-regulated in tumors in GSE16515 dataset and *CRP* in GSE15471. Interestingly, *AZGP1 is* significantly down-regulated in tumors in both datasets. The other secreted protein genes are not significantly regulated in pancreatic tumor samples (Fig. 1e).

### IGFBP-3 is transcriptionally increased in pancreatic tumor

Previous studies have indicated that Hippo-induced cancer-like cell over-proliferation leads to muscle dysfunction and systemic tissue wasting via production of ImpL2, an insulin-like binding protein, and inhibition of insulin-like signaling in fly model [14]. To address whether the mechanism is conserved in pancreatic cancer cachexia, we measured the expression levels of human ImpL2 homologs in pancreatic tumors. Six structural homologs of ImpL2, IGFBP 1–6, and one functional homolog, IGFBP-7, exist in human [16, 30]. Interestingly, both *IGFBP3* (two probes, more than eight folds) and *IGFBP5* (three probes, more than 3 folds) are dramatically increased in both pancreatic tumor datasets, whereas, *IGFBP2* is significantly decreased in both datasets (Fig. 1e). *IGFBP1* (more than five folds) is induced only in GSE16515 and *IGFBP 4 & 7* (more than three & five folds) are induced only in GSE15471 dataset (Fig. 1e). As the *IGFBP3* exhibited the highest induction in both pancreatic tumor datasets, we proposed that pancreatic tumors probably communicate to muscle cells via production of IGFBP-3.

### IGFBP-3 impairs C2C12 myogenesis

**Fig. 2:**
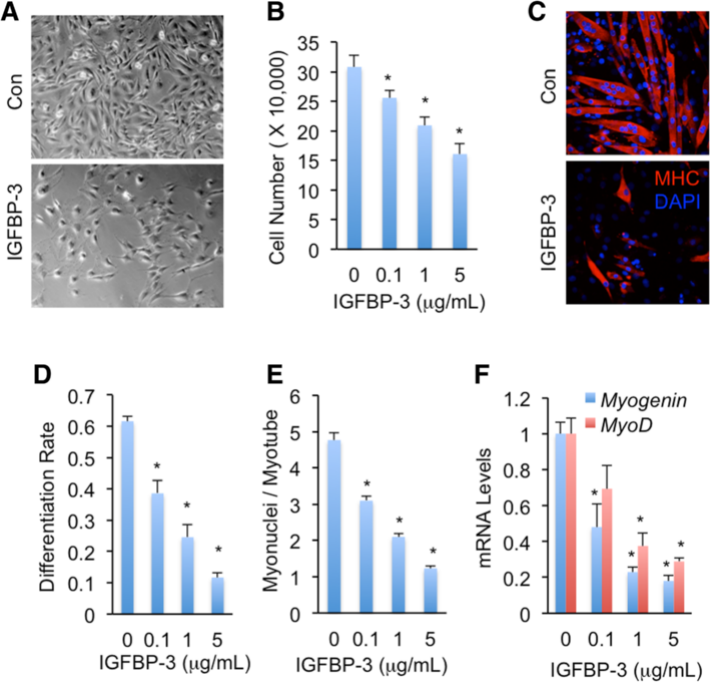
IGFBP-3 impairs C2C12 myoblast proliferation and C2C12 myotube differentiation. **a**-**b** C2C12 myoblasts were seeded at density of 20,000 cells/well and cultured in growth medium with or without different doses of IGFBP-3 for 96 h (**a**) and cells were counted (**b**). **c** C2C12 myoblasts were differentiated with or without 5 μg/mL IGFBP-3 for 96 h. Differentiated myotubes were indicated as MHC-positive cells. **d**-**f** Myotube differentiation rate indicated by percentage of nuclei in MHC-positive cells (**d**), Myonuclei number per myotube (**e**), and mRNA expression levels indicated by qPCR of muscle regulatory factor *MyoD* and *Myogenin* (**f**) were measured after differentiation with different doses of IGFBP-3 treatment for 96 h. Data are presented as means ± SEM. * *p* < 0.05

Increasing evidence indicated that impaired myogenesis, including myoblast proliferation and myotube differentiation, contributes to muscle wasting [4–6, 31]. Excess IGFBP5 has been shown to regulate myotube differentiation probably via modulation of IGF-2 signaling [19, 20], however, whether IGFBP-3, which predominantly binds to IGF-1 and exhibits the greatest induction in pancreatic tumors, affects myogenesis is largely unknown. To address this question, we studied whether IGFBP-3 regulates myoblast proliferation and myotube differentiation. We incubated C2C12 myoblasts with different doses of synthetic IGFBP-3 and measured the myoblast proliferation rate. Interestingly, IGFBP-3 significantly inhibited C2C12 myoblast proliferation, which was indicated by reduced myoblast cell number, in a dose-dependent manner (Fig. 2a-b). Then we incubated C2C12 myoblasts with different doses of synthetic IGFBP-3 from day 0 (D0) of differentiation and monitored myotube differentiation rate. Impressively, IGFBP-3 treatment potently reduced the number of MHC-positive differentiated myotubes in a dose-dependent manner on day 4 (D4) of differentiation as compared with untreated controls (Fig. 2c). The differentiation rate indicated by cell morphology and the fusion rate indicated by nuclei number per myotube were also dramatically decreased by IGFBP-3 treatment (Fig. 2d-e). We also measured the expression of muscle regulatory factors MyoD (myogenic differentiation 1) and Myogenin. On the D4 of differentiation, IGFBP-3 treatment significantly decreased mRNA levels of both *MyoD* and *Myogenin* (Fig. 2f). Collectively, our results indicated that IGFBP-3 impairs C2C12 myogenesis.

### IGFBP-3 promotes C2C12 myotube protein degradation

**Fig. 3:**
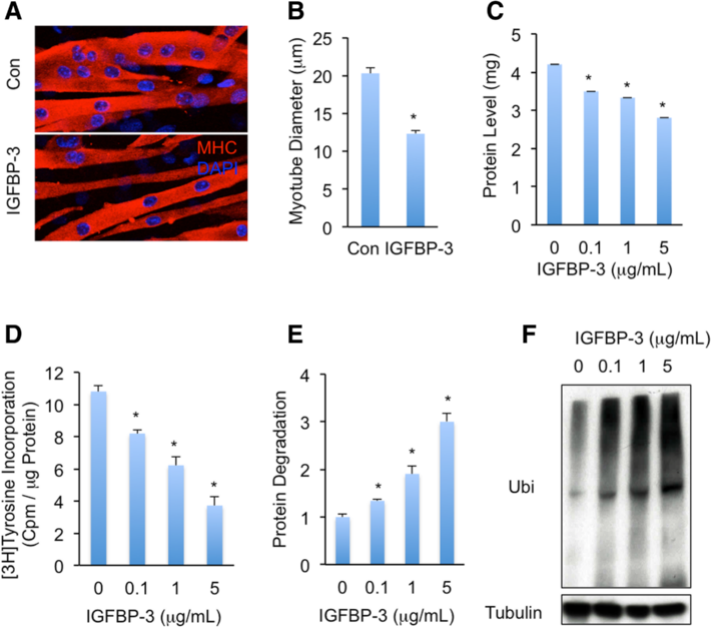
IGFBP-3 enhances C2C12 myotube protein degradation. **a**-**b** C2C12 myotubes, which were differentiated for 96 h and were indicated as MHC-positive cells (**a**), were treated with or without 5 μg/mL IGFBP-3 for 48 h and myotube diameters (**b**) were measured to assess myotube atrophy phenotype. **c**-**f** Protein levels in 6-well plate wells (**c**), protein synthesis (**d**), protein degradation (**e**), and level of ubiquitinated proteins (**f**) of C2C12 myotubes that were differentiated for 96 h and treated with different doses of IGFBP-3 for 48 h. Data are presented as means ± SEM. * *p* < 0.05

The atrophy of skeletal muscle due to the elevated activity of proteolytic pathways and protein degradation is another important feature of cancer-induced muscle wasting [32]. To examine whether IGFBP-3 induces muscle wasting in myotube, we treated C2C12 myotubes on day 4 (D4) of differentiation with different doses of IGFBP-3 for 48 h. Impressively, IGFBP-3 treatment resulted in a distinct atrophic myotube phenotype (Fig. 3a) with a decrease in myotube diameter (Fig. 3b). IGFBP-3-induced atrophy was also evidenced by the loss of myotube protein in a dose-dependent manner (Fig. 3c). Moreover, the amount of protein synthesis that was quantified with [^3^H] tyrosine incorporation was potently reduced after 24 h IGFBP-3 treatment (Fig. 3d). In contrast, the level of protein degradation indicated by [^3^H] tyrosine loss after 24 h IGFBP-3 treatment was significantly elevated as compared to untreated controls (Fig. 3e), indicating that the proteolysis highly associated with myotube wasting is enhanced by IGFBP-3 treatment. The enhanced proteolysis could be caused by elevated activity of ubiquitin-proteasome pathway [4, 33]; to assess this possibility, the amount of ubiquitinated protein after IGFBP-3 treatment was measured. Consistent to our hypothesis, immunoblot analysis using ubiquitin antibody revealed that IGFBP-3 increased protein ubiquitination in myotube in a dose-dependent manner (Fig. 3f). Thus, our results indicated that IGFBP-3 enhances ubiquitination associated proteolysis and reduces C2C12 myotube mass.

### IGFBP-3 induces muscle-wasting response in myotubes via suppression of IGF-1/PI3K/AKT signaling

**Fig. 4:**
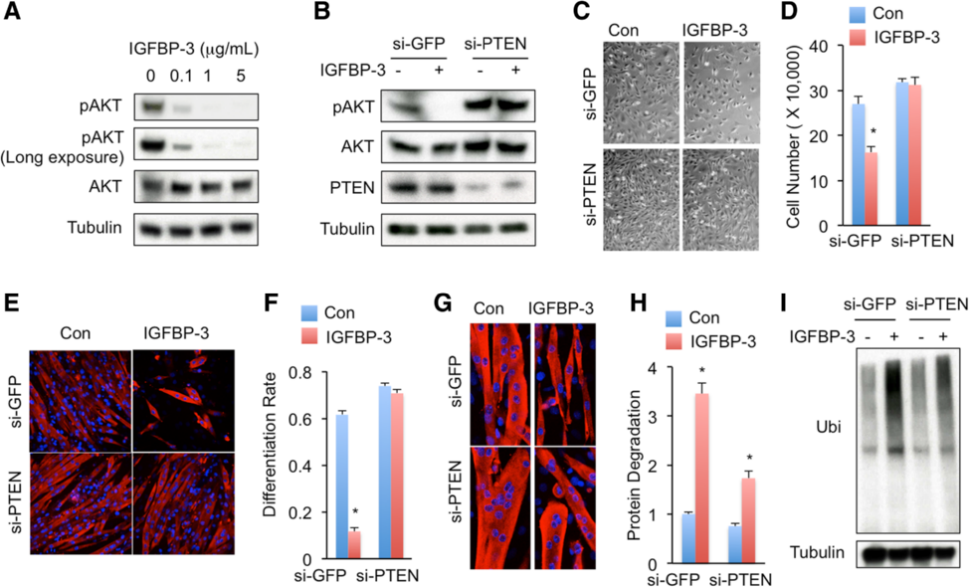
IGFBP-3 regulates muscle wasting via inhibition of IGF signaling. **a** IGF signaling indicated as p-AKT level in normal C2C12 myoblasts that were treated with different doses of IGFBP-3 for 24 h. **b** IGF signaling in myoblasts that were infected with si-GFP (control) or si-PTEN lentivirus for 48 h and were treated with or without 5 μg/mL IGFBP-3 for 24 h. **c**-**d** Myoblasts were seeded at density of 20,000 cells/well and were grown with or without 5 μg/mL IGFBP-3 for 96 h (**c**) and cells were counted (**d**). **e**-**f** C2C12 myotubes were differentiated with or without 5 μg/mL IGFBP-3 for 96 h (**e**) and differentiation rates (**f**) were measured then. **g**-**i** C2C12 myotubes were differentiated for 96 h and treated with 5 μg/mL IGFBP-3 for 48 h. The myotube atrophy phenotype (**g**), degraded protein (**h**) and ubiquitinated protein levels (**i**) were measured. Data are presented as means ± SEM. * *p* < 0.05

IGFBP-3 is the major binding protein for IGF-1 (>80 %) and circulates throughout the body to control IGF-1 access to cell-surface receptors and downstream PI3K/AKT signaling [16]. In addition, IGF-1/PI3K/AKT signaling has been shown to promote myogenesis and myotube hypertrophy [17, 34]. We hypothesized that IGFBP-3 regulates myogenesis and myotube protein degradation via modulation of PI3K/AKT signaling. We next asked whether exogenous IGFBP-3 inhibits PI3K/AKT signaling in myotubes. C2C12 myoblasts were exposed to IGFBP-3 and PI3K/AKT signaling was measured. Immunoblot analysis indicated that phosphorylation of AKT, the major readout of IGF-1 signaling, was dramatically decreased by IGFBP-3 treatment, at least at 0.1 μg/mL concentration (Fig. 4a), demonstrating that exogenous IGFBP-3 blocked PI3K/AKT signaling in muscle cells. To verify whether suppressed PI3K/AKT signaling contributes to impaired myogenesis and myotube wasting, we blocked PI3K/AKT signaling using chemical inhibitor LY294002 that potently suppresses PI3K activity [35]. Immunoblot analysis indicated that, similar to IGFBP-3 treatment, LY294002 dramatically inhibited AKT phosphorylation (Additional file 4: Figure S2A). Importantly, compared to IGFBP-3, LY294002 showed a much stronger inhibition on myoblast proliferation and myotube differentiation, including decreased MHC-positive cells, myonuclei number, as well as *Myogenin* and *MyoD* expression levels (Additional file 4: Figure S2B-S2G and Additional file 5: Figure S6B). We subsequently tested the effect of LY294002 on myotube wasting. Consistently, LY294002 treatment potently resulted in myotube atrophy and enhanced protein ubiquitination (Additional file 4: Figure S2H-S2I, Additional file 5: Figure S6B, and Additional file 6: Figure S7B).

We also asked whether IGFBP-3 attenuates IGF-1 effects on myogenesis and myotube wasting. Consistent with previous reports [17, 34], IGF-1 significantly augmented IGF signaling in C2C12 myoblast, indicated by AKT phosphorylation, C2C12 myoblast proliferation and myotube differentiation, as well as myotube wasting (Additional file 7: Figure S3). Interestingly, IGFBP-3 suppressed IGF-1-induced PI3K/AKT signaling in a dose-dependent manner (Additional file 7: Figure S3A). Moreover, IGFBP-3 potently attenuated IGF-1-induced C2C12 myoblast proliferation, myotube differentiation, and myotube protein degradation (Additional file 7: Figure S3B-S3H).

Taken together, our results indicated that IGFBP-3 impairs C2C12 myogenesis and promotes C2C12 myotube wasting via suppression of IGF-1/PI3K/AKT signaling.

### Enhanced PI3K/AKT signaling completely rescues IGFBP-3-induced C2C12 myogenesis inhibition, but partially rescues IGFBP-3-induced C2C12 myotube wasting

To test whether IGFBP-3 inhibits myogenesis in a PI3K/AKT-signaling-dependent manner, we manipulated PI3K/AKT signaling upon IGFBP-3 treatment. PTEN is an established suppressor of PI3K/AKT signaling. Loss of PTEN expression or activity has been shown to enhance PI3K/AKT signaling in multiple tissues [36–38]. Lentivirus-mediated PTEN loss, which was detected by immunoblot, significantly enhanced PI3K/AKT signaling and completely abolished IGFBP-3 inhibitory effect of AKT phosphorylation (Fig. 4b). Importantly, enhanced PI3K/AKT signaling also completely restored myoblast proliferation and myotube differentiation, including MHC-positive cells, myonuclei number, as well as *Myogenin* expression level, even under IGFBP-3 treatment (Fig. 4c-f, Additional file 8: Figure S4, and Additional file 5: Figure S6A). Taken together, our results indicated that IGFBP-3 inhibits myogenesis in a PI3K/AKT-signaling-dependent manner.

We next examined whether elevated PI3K/AKT signaling is sufficient to rescue IGFBP-3-induced myotube wasting. PTEN expression was knocked down via lentivirus infection in myotubes and atrophy phenotype was monitored. Surprisingly, even though PTEN knockdown improved myotube atrophy phenotype at basal level, PTEN knockdown only partially rescued IGFBP-3-induced myotube atrophy (Fig. 4g and Additional file 6: Figure S7A). We further measured the ubiquitin-proteasome pathway. Consistent to myotube atrophy phenotype, PTEN loss partially rescued IGFBP-3-induced protein degradation and protein ubiquitination (Fig. 4h-i). Collectively, IGFBP-3 promotes C2C12 myotube wasting via PI3K/AKT signaling and other unknown signals.

### IGFBP-3 is required for Capan-1 pancreatic cancer cells to induce C2C12 myotube wasting

**Fig. 5:**
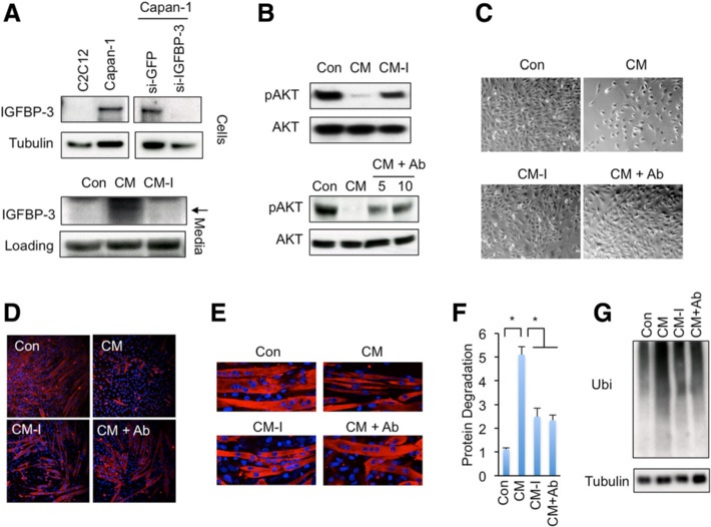
IGFBP-3 is required for Capan-1 cells to induce muscle wasting. **a** IGFBP-3 protein levels in normal C2C12 myoblasts, normal Capan-1 cells, and Capan-1 cells infected with si-GFP or si-IGFBP-3 lentivirus for 48 h were indicated by immunoblot (Up). IGFBP-3 levels in normal growth medium (Con) and conditioned medium of Capan-1 cells infected with si-GFP (CM) or si-IGFBP-3 (CM-I) lentivirus were indicated by immunoblot (Down, non-specific band of IGFBP-3 antibody were used as loading control). **b** IGF signaling, which is indicated with pAKT levels, in C2C12 myoblasts that were treated with normal DMEM growth medium (Con) or conditioned growth medium from si-GFP (CM) or si-IGFBP3 (CM-I) Capan-1 cells for 24 h (UP); or with Con, CM, or CM that was pre-treated with 5 μg/mL or 10 μg/mL IGFBP-3 antibody for 30 min (CM + Ab) for 24 h. **c** Myoblasts were seeded at density of 20,000 cells/well and were grown with Con, CM, CM-I, or CM + Ab (10 μg/mL IGFBP-3 antibody) growth medium for 96 h. **d** Myotubes were differentiated in Con, CM, CM-I, or CM + Ab (10 μg/mL IGFBP-3 antibody) differentiation medium for 96 h. **e**-**g** C2C12 myotubes were normally differentiated for 96 h and then incubated with Con, CM, CM-I, and CM + Ab (10 μg/mL IGFBP-3 antibody) differentiation medium for 48 h. The protein degradation (**f**) and ubiquitinated protein levels (**g**) were measured. Data are presented as means ± SEM. * *p* < 0.05

Treating muscle cells or adipocytes with conditioned medium from pancreatic cancer cells was used to mimic tumor-host interaction and identify potential tumor-derived secret factors. Capan-1 pancreatic cancer cells have been established to cause muscle wasting [10, 11]. We next assessed whether pancreatic cancer cells induce myotube wasting via IGFBP-3 production. We first measured the IGFBP-3 level in Capan-1 pancreatic cancer cells. Immunoblot analysis with a specific antibody against IGFBP-3 revealed that, compared to C2C12 myoblasts, Capan-1 cells produced abundant IGFBP-3 (Fig. 5a, up). Similarly, abundant IGFBP-3 was detected in conditioned medium from Capan-1 cells, but not in normal growth medium (Fig. 5a, down). Subsequently, compared to normal growth medium (Con), conditioned growth medium from Capan-1 cells (CM, see Methods) dramatically decreased IGF signaling in myoblasts (Fig. 5b) and inhibited myoblast proliferation (Fig. 5c and Additional file 9: Figure S5B). In addition, conditioned differentiation medium from Capan-1 cells (CM) inhibited myotube differentiation (Fig. 5d, Additional file 9: Figure S5C-S5E and Additional file 5: Figure S6C) and promoted myotube protein degradation and atrophy (Fig. 5e-g, Additional file 9: Figure S5F and Additional file 6: Figure S7C) compared to normal differentiation medium (Con).

We further examined whether removal of IGFBP-3 is able to attenuate muscle-wasting effect in myotubes. Lentivirus-induced RNAi dramatically decreased IGFBP-3 levels in both Capan-1 cells and conditioned medium (Fig. 5a and Additional file 9: Figure S5A). On the other hand, we also neutralized IGFBP-3 by pre-incubating Capan-1 conditioned medium with IGFBP-3 antibody. Strikingly, compared to CM, both conditioned growth medium from IGFBP-3-knockdown Capan-1 cells (CM-I) and Capan-1 conditioned growth medium that was pre-treated with IGFBP-3 antibody (CM + Ab) significantly restored p-AKT levels in C2C12 myoblasts (Fig. 5b). Moreover, CM-I and CM + Ab growth medium both alleviated CM-induced inhibition of myoblast proliferation (Fig. 5c and Additional file 9: Figure S5B). Both CM-I and CM + Ab differentiation medium also alleviated CM-associated myotube differentiation inhibition, myotube protein degradation, and myotube atrophy (Fig. 5d-g, Additional file 9: Figure S5C-S5F, Additional file 5: Figure S6C and Additional file 6: Figure S7C).

Capan-1 cells produce multiple secreted proteins that might contribute to myotube wasting, thus our results indicated that pancreatic cancer cells induce myotube wasting via, at least, IGFBP-3 secretion.

### Capan-1 pancreatic cancer cells can escape from IGFBP-3 inhibitory effects

**Fig. 6:**
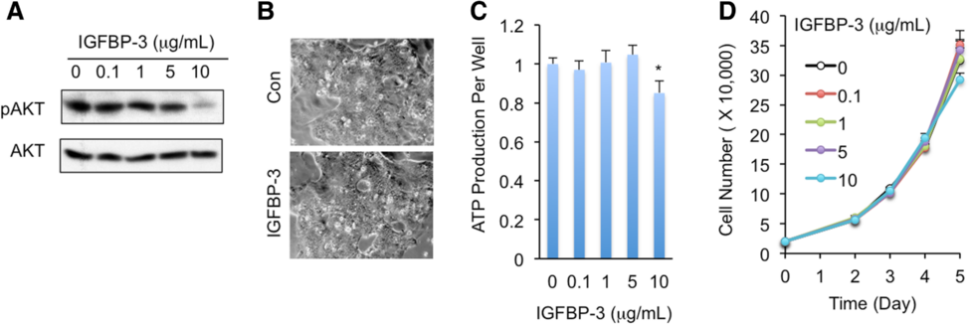
Capan-1 cells escape from IGFBP-3 inhibitory effects. **a** IGF signaling indicated as p-AKT level in Capan-1 cells that were treated with different doses of IGFBP-3 for 24 h. **b**-**d** Capan-1 cells were seeded at density of 20,000 cells/well and treated with 5 μg/mL IGFBP-3 for 120 h (**b**). ATP levels per well (**c**) and cells (**d**) were measured when Capan-1 cells were treated with different doses of IGFBP-3 for 120 h. Data are presented as means ± SEM. * *p* < 0.05

Previous *Drosophila* studies indicated that cancer-like cells restrain host tissue growth via ImpL2 production, but these cancer-like cells escape from ImpL2 inhibitory effects [14]. We next examined whether Capan-1 pancreatic cancer cells escape from IGFBP-3 inhibition in a similar manner. We treated Capan-1 cells with different doses of IGFBP-3 and monitored IGF signaling and cell proliferation. Strikingly, in contrast to the fact that IGF signaling in C2C12 myoblast was significantly suppressed by IGFBP-3 at 0.1 μg/mL (Fig. 4a), IGF signaling in Capan-1 cells wasn’t suppressed by IGFBP-3 unless at a very high dose, namely 10 μg/mL (Fig. 6a), suggesting that the sensitivity to IGFBP-3 in Capan-1 cancer cells is decreased by over 100 folds compared to C2C12 myoblasts. Furthermore, IGFBP-3 below 10 μg/mL failed to restrain ATP production, which is indicative of cancer cell proliferation, and to change the growth rate of Capan-1 cells (at all concentrations tested) (Fig. 6b-d). This result was also in strong contrast with the fact that 0.1 μg/mL IGFBP-3 is sufficient to impair C2C12 myogenesis and induce C2C12 myotube wasting. Thus, our results indicated that Capan-1 pancreatic cancer cells escape from IGFBP-3 inhibitory effects.

## Discussion and conclusions

Pancreatic cancer patients have the highest prevalence and often develop the most severe degrees of cachexia including muscle wasting. One of the main pathogenic mechanisms underlying cancer-induced muscle wasting is the tumor-host interaction, thus examining the secreted proteins that are specifically produced in tumor cells and regulate muscle function will provide novel insights in the understanding of pancreatic cancer cachexia. We performed bioinformatics analyses in two different gene expression datasets of pancreatic ductal adenocarcinoma and mined the secreted protein genes that are dramatically induced in pancreatic tumors. Genes, which encode systemic inflammatory factors, including *CRP*, *ILs*, as well as *TNF* [9, 24], are not consistently up-regulated in pancreatic tumors. This is consistent with the notion that these inflammatory factors are predominantly produced in host tissues. Similarly, our results also indicated that other cachexia-associated genes, like *DCD*, *AZGP1*, *GDF15*, and *MSTN* [4, 25, 28, 29], are not predominantly produced in pancreatic tumors. They are mainly produced in liver, adipose tissue, and muscle. *TGFB1* and *TGFB2,* which encode TGF-β1 and TGF-β2, respectively, are moderately elevated in pancreatic tumors (~3 folds, data not shown). Our results suggested that pancreatic tumor-derived TGF-βs may contribute to cachexia and muscle wasting, even though a few studies indicated that functional TGF-βs are produced in multiple host tissues [39, 40]. Importantly, *INHBA* that encodes activin A and activin BA is dramatically increased in pancreatic tumors (>10 folds) in both datasets. As activin A is highly associated with cancer cachexia [26], our results indicated that *INHBA* induction in pancreatic tumors might play a critical role in cancer-associated cachexia. Another tumor-derived secreted protein gene *PTHLH*, which encodes PTH-related protein and induces muscle wasting [12], is up-regulated only in GSE16515 dataset. Thus, our results demonstrated that the secreted protein genes induced in pancreatic tumors are valuable in uncovering tumor-derived factors causing muscle wasting and cancer cachexia.

IGFBPs homolog, ImpL2, has been shown to be produced in cancer-like cells and to impair host tissue growth and muscle function via inhibition of insulin-like signaling in *Drosophila* model [14, 41]. In this study we measured the expression patterns of all *IGFBPs* in two different datasets to validate the conserved regulation in pancreatic tumor samples. Interestingly, expression levels of *IGFBP3* (>8 folds) encoding IGFBP-3 were dramatically induced in both pancreatic tumor datasets. The induction of *IGFBP3* in pancreatic tumor samples was also observed in previous studies [42, 43]. In addition, we also found that IGFBP-3 is abundantly produced in Capan-1 pancreatic cancer cells and secreted into culture medium. We further demonstrated that either exogenous IGFBP-3 or IGFBP-3-enriched Capan-1 cell-conditioned medium potently enhances muscle wasting via both impaired C2C12 myogenesis and increased C2C12 myotube proteolyisis. Strikingly, IGFBP-3 deprivation in Capan-1 cell-conditioned medium, which is achieved by knockdown of IGFBP-3 expression in Capan-1 cells or specific IGFBP3 antibody neutralization, significantly improved the wasting effects in muscle cells. Thus, our results indicated that pancreatic cancer cells directly cause muscle cell wasting via IGFBP-3 production.

IGF-1 signaling stimulates muscle growth and protein synthesis, as well as proliferation and differentiation of satellite cells, and exerts anti-apoptotic effects on muscle cells to suppress proteolysis and inhibit the ubiquitin-proteasome system [17, 44–46]. IGFBP-3 has been shown to bind to IGF-1 and IGF-1/IGFBP-3 ratio in serum is essential for IGF-1 bio-ability and IGF-1 signaling [16]. Moderate amount of IGFBP-3 promotes IGF-1 stability in blood and its interaction with IGF1 receptor, as well as intracellular IGF-1 signaling. Injection of IGF-1/IGFBP-3 complex, not IGFBP-3 alone, into tumor-bearing mice attenuates cancer cachexia phenotypes, including weight loss and appetite, probably due to stabilized IGF-1 in blood and enhanced IGF-1 signaling [23]. However, excess IGFBP-3 prevents IGF-1 from binding to its receptor and inhibits IGF-1 signaling. In this study, we observed that both exogenous IGFBP-3 and IGFBP-3-enriched Capan-1 conditioned medium potently decrease IGF-1 signaling in muscle cells. Our results indicated that Capan-1 pancreatic cancer cells produce IGFBP-3 to restrain IGF-1 availability, suppress IGF-1/PI3K/AKT signaling in muscle cells, and induce muscle cell wasting.

Another important finding of our study is that we uncoupled IGFBP-3 mechanistic impacts on myogenesis and myotube protein degradation. Genetic enhancement of PI3K/AKT signaling managed to rescue IGFBP-3 inhibition of myogenesis, including C2C12 myoblast proliferation and C2C12 myotube differentiation; however, it failed to fully rescue IGFBP-3-induced C2C12 myotube proteolysis. It is possible that regulation of myotube proteolysis is much more complicated than myogenesis regarding IGFBP-3 function. IGF signaling controls myotube proteolysis via two important regulators downstream PI3K/AKT cascade, FoxO1 and mTOR [17, 47]. AKT phosphorylates FoxO1 and suppresses its transcriptional activity of ubiquitin ligases expression to inhibit proteolysis [17]. AKT also phosphorylates mTOR to promote protein synthesis via activation of 4E-BP1 and S6K [47]. It is well known that, in addition to IGF signaling, IGFBP-3 also triggers other pathways, like TGF-β, NF-κB, and JAK/STAT signalings [21, 48–50], in a cell-context manner. Thus, we speculated that IGFBP-3 enhances myotube proteolysis via IGF-signaling-independent mechanisms as well. How other pathways are involved in IGFBP-3 regulation of myotube proteolysis will be another interesting question for our future research.

Several cytokines/hormones have been reported to modulate tumor cell growth in an autocrine manner [51–53]. However, in this study we indicated that growth rate of Capan1 pancreatic cancer cells is rarely affected by IGFBP-3. Another outstanding question in this field could be: how do pancreatic tumor cells survive while other host tissue growths are suppressed by IGFBPs? Previous studies mentioned that tumors or cancer-like cells survive from IGFBPs suppression 1) overexpressing IGF signaling components, like IGF1R, PI3K, as well as AKT, to potentiate intracellular IGF signaling; 2) overexpressing other growth signaling components, like EGFR, JAK/STAT, as well as TGF-β pathways, to compensate IGF growth signaling [14, 16]. A plausible explanation is that, similar to *Drosophila* Yki-induced cancer-like cells [14], pancreatic cancer cells obtain enhanced IGF signaling independent of extracellular IGF1/IGFBP-3 impact. In addition, our bioinformatics analysis uncovered that genes encoding components of TGF-β, integrin, p53, and PDGF signaling pathways, are dramatically increased and significantly enriched in both pancreatic tumor datasets, suggesting that pancreatic tumor cells increase other growth signalings to counteract IGFBP-3 suppression.

## Methods

### Bioinformatics analyses of microarray results

Microarray data GSE16515 and GSE15471 were obtained from NCBI-GEO (www.ncbi.nlm.nih.gov/geo). Expression levels of probes were normalized using RMA and mapped in Affymetrix Human Genome U133 Plus 2.0. Gene expressions in pancreatic cancer samples with fold change > =2 and false discovery rate (FDR) < 0.05 were considered as significant. All comparisons were made between pancreatic cancer tissues and normal tissues. As for gene ontology enrichment analysis, the significantly up- and down-regulated genes were uploaded separately to DAVID Bioinformatics Resource [54]. The human genome U133 Plus was used as a background for the GO analysis. The GO terms with EASE score < 0.05 were selected for interpretation [55]. After finding overlapping genes/probes of datasets GSE15471 and GSE16515, Pearson's correlation analysis of fold change of each detected gene in each dataset was performed. Predicted secreted protein genes were annotated regarding gene lists that encode secreted proteins from both The Human Protein Atlas and (www.proteinatlas.org) and GeneCards (www.genecards.org). Heatmaps of selected genes were made using ggplot2 package of software R.

### Antibodies and reagents

Anti-MHC (MF20) and α-tubulin antibodies were obtained from the Developmental Studies Hybridoma Bank and Sigma, respectively. Anti-PTEN, phospho-AKT (S473), AKT and ubiquitin antibodies were purchased from Cell Signaling Technology. Anti-IGFBP-3 antibody was purchased from Santa Curz Biotechnology. All secondary antibodies for immunostaining and western blot were from Jackson Laboratory and Thermo Fisher Scientific, respectively. Recombinant human IGFBP-3 and IGF-1 proteins were obtained from R&D Systems. PI3K inhibitor LY294002 was from Abcam. shRNA lentivirus vectors for *PTEN* (TRCN0000002747) and *IGFBP3* (TRCN0000072512) were purchased from Sigma.

### Cell culture and treatment

Murine C2C12 myoblasts obtained from American Type Culture Collection (ATCC) were maintained in Dulbecco’s modified Eagle’s medium (DMEM) supplied with 10 % fetal bovine serum and antibiotics (50 U/ml penicillin and 50 μg/ml streptomycin), referred as growth medium. To induce myogenic differentiation, myoblasts were grown to reach 100 % confluence (day 0) and cultured with DMEM containing antibiotics and 2 % heat-inactivated horse serum, referred as differentiation medium. Capan-1 pancreatic cancer cells were obtained from ATCC and cultured in ATCC-formulated Iscove's Modified Dulbecco's Medium with 20 % fetal bovine serum and antibiotics. For CM (conditioned medium) preparation, Capan-1 cells were plated at a density of 50,000 cells/cm^2^, and after 12 h of seeding, cells were washed twice with PBS and cultured in growth or differentiation medium for C2C12 cells for the next 24 h. Conditioned mediums were centrifuged at 1200 g for 10 min and filtered with a 0.2 μm syringe filter and used immediately or stored at −80 °C. For the neutralization of IGFBP-3 in CM, anti-IGFBP-3 antibody was pre-incubated at five and 10 μg/mL in CM for 30 min. For IGFBP-3 treatment, myoblasts or myotubes were treated with indicated amounts of IGFBP-3 in growth medium or differentiation medium.

### Cell proliferation assays

C2C12 myoblasts and Capan-1 cells were seeded at a density of 20,000 cells/well with growth medium. Cells were counted at different time points after treatment of indicated reagents. Total ATP level in cells per well was determined by using an ATP assay kit (Roche).

### Immunostaining and myotube analysis

For immunofluorescence analysis, cells were seeded onto sterile preprocessed glass coverslips that were pre-coated with 1 % gelatin. After the differentiation to myotubes, cells were washed two times with PBS followed by fixation in 4 % paraformaldehyde for 15 min. After being rehydrated in PBS, cells were blocked for 30 min in 1 % Bovine serum albumin (BSA) in PBST, PBS containing 0.2 % Triton-X. Afterward, cells were incubated with anti-MHC (1:20) in 1 % BSA/PBST overnight in cold room. Cells were next incubated with fluorescence labeled secondary anti-mouse antibody (1:200) and DAPI (1:1000) at room temperature for 1 h. The specimens were examined in a Leica TCS-NT laser scanning confocal microscope. The myotube differentiation index was calculated as the percentage of nuclei in MHC-positive myotubes. The nuclei number in each MHC-positive myotube was also calculated to evaluate differentiation. To assess myotube atrophy, diameters of myotubes were measured for each condition separated by 50 μm along the length of the myotube. Each data point was generated from at least 200 randomly chosen MHC-positive myotubes.

### Western blot

Cell cultures were rinsed in PBS and lysed in 50 mM Tris–HCl, pH 7.2, 150 mM NaCl, 1 % Nonidet P-40, and 1 % protease and phosphatases inhibitor mixture (Sigma), followed by a 10-min high-speed centrifugation for the collection of lysis supernatant. Individual proteins were separated in SDS-PAGE gel and transferred into nitrocellulose membrane. Membranes were probed with indicated primary antibodies (p-AKT, 1:1000; AKT, 1:1000, α-tubulin, 1:10,000. PTEN, 1:1000. IGFBP-3, 1:500). For detection, anti-rabbit or anti-mouse HRP-conjugated secondary antibodies were used followed by visualization with ECL. Representative western blotting images of multiple independent biological experiments were presented.

### Quantitative RT-PCR

Total RNAs extracted from cells were reverse transcribed using oligo dT primers and quantitative PCR assays of cDNA pools were carried out using the CFX96 Real-time PCR system (Bio-Rad) to evaluate the abundance of target transcripts in relative to house-keeping gene GAPDH. Target cDNAs were amplified using the following probe sets.

MyoD-sense: TACAGTGGCGACTCAGATGC

MyoD-antisense: GAGATGCGCTCCACTATGCT

Myogenin-sense: CTACAGGCCTTGCTCAGCTC

Myogenin-antisense: ACGATGGACGTAAGGGAGTG

IGFBP3-sense: GTGTACTGTCGCCCCATCCC

IGFBP3-antisense: CTCGCAGCGCACCACG

GAPDH-sense: TGCGACTTCAACAGCAACTC

GAPDH-antisense: GCCTCTCTTGCTCAGTGTCC

### Lentivirus-mediated RNAi

shRNAs in the pLKO.1-puromyosin vector were used for knocking down PTEN or IGFBP3 expression. Lentivirus packaging and testing were performed as previously described [56]. C2C12 or Capan-1 cells were infected with lentivirus in medium containing 8 μg/ml polybrene and selected in 3 μg/ml puromycin for 4 days. Knockdown efficiency was further confirmed with western blot or qPCR.

### Measurement of protein synthesis and degradation in C2C12 myotubes

Total protein synthesis was assessed quantifying the amount of [^3^H] tyrosine (PerkinElmer) incorporation into C2C12 myotube cultures. After treatment of indicated reagents for 24 h, the C2C12 myotubes were incubated with differentiation medium containing 5 μCi/ml [^3^H] tyrosine for 2 h. The medium then was discarded, cells were washed twice with PBS, and 1 mL of 10 % trichloroacetate was added to each well. Total cell lysates were centrifuged and the pellets were washed with 95 % ethanol and dissolved in 0.1 M NaOH. Samples were analyzed for total radioactivity and protein concentration using a scintillation counter (PerkinElmer) and the Bradford assay, respectively. The final radioactivity was normalized to protein level. As for protein degradation, C2C12 myotubes were incubated with 5 μCi/ml [^3^H] tyrosine for 48 h to label cellular proteins. Afterward, myotubes were incubated with medium containing 2 mM unlabeled tyrosine and indicated reagents control for 24 h. The medium was collected, precipitated with 10 % trichloroacetate and centrifuged. The acid-soluble radioactivity, which reflects degraded protein level, was measured using a scintillation counter.

### Statistical analysis

Statistical analysis was performed using Student’s *t* test in Microsoft Excel. The values were presented as means ± SEM and significance was defined as * *P* < 0.05.

## Additional files

Additional file 1: Figure S1. Gene ontology enrichment analysis of differentially expressed genes in pancreatic tumors compared to normal tissues. (**A**) Significantly down-regulated genes (fold change > = 2, FDR < 0.05) were identified in two pancreatic tumor microarray datasets. The number of the overlapping genes was indicated in the middle. (**B**-**C**) Pearson’s correlation analysis of fold change (pancreatic tumor/normal tissue) of overlapping up-regulated (**B**) and down-regulated (**C**) genes in each pancreatic tumor dataset. (**D**-**E**) Significantly enriched (EASE score < 0.05) GO terms were identified from the overlapping differentially expressed genes (**D**, signaling pathways for up-regulated genes. **E**, biological process for down-regulated genes). (TIFF 6076 kb) Additional file 2: Table S1. Bioinformatics analysis results: differentially expressed genes in GSE15471 and GSE16515. (XLSX 4326 kb) Additional file 3: Table S2. Overlap of differentially expressed genes in datasets GSE15471 and GSE16515 and retrieval of upregulated secreted proteins in databases "Human Atlas" and "Genecards". (XLSX 50 kb) Additional file 4: Figure S2. Impaired IGF signaling inhibits C2C12 myogenesis and induces C2C12 myotube protein degradation. (**A**) IGF signaling indicated as p-AKT level in C2C12 myoblasts that were treated with or without 10 μM LY294002 for 24 h. (**B**-**C**) Myoblasts were seeded at density of 20,000 cells/well and were grown with or without 10 μM LY294002 for 96 h (**B**) and cells were counted (**C**). (**D**-**G**) C2C12 myotubes were differentiated with or without 10 μM LY294002 for 96 h (**D**) and differentiation rate (**E**), myonuclei number (**F**), as well as muscle regulatory factors expression (**G**) were measured then. (**H**-**I**) C2C12 myotubes were differentiated for 96 h and treated with 10 μM LY294002 for 48 h (**H**). The ubiquitinated protein levels were measured (**I**). Data are presented as means ± SEM. * *p* < 0.05. (TIFF 6076 kb) Additional file 5: Figure S6. IGF signaling, IGFBP-3, and conditioned medium from Capan-1 cells regulate C2C12 myotube differentiation. C2C12 myotube differentiation status. (**A**) si-GFP and si-PTEN myoblasts were differentiated with or without 5 μg/mL IGFBP-3 from Day 0 to Day 4. (**B**) Normal myoblasts were differentiated with or without 10 μM LY294002 for 96 h (Day 4). (**C**) Normal myoblasts were differentiated in Con, CM, CM-I, or CM + Ab (10 μg/mL IGFBP-3 antibody) differentiation medium for 96 h (Day 4). (TIFF 6076 kb) Additional file 6: Figure S7. IGF signaling, IGFBP-3, and conditioned medium from Capan-1 cells regulate C2C12 myotube atrophy. C2C12 myotube atrophy phenotype. (**A**) si-GFP and si-PTEN myoblasts were differentiated for 96 h and then treated with or without 5 μg/mL IGFBP-3 for 48 h. (**B**) Myotubes were differentiated for 96 h and then treated with or without 10 μM LY294002 for 48 h. (**C**) Myotubes were normally differentiated for 96 h and then moved to Con, CM, CM-I or CM + Ab (10 μg/mL IGFBP-3 antibody) differentiation medium for 48 h. (TIFF 6076 kb) Additional file 7: Figure S3. IGFBP-3 counteracts IGF-1 effects on C2C12 myogenesis and C2C12 myotube protein degradation. (**A**) C2C12 myoblasts were deprived from serum overnight and then treated with different doses of IGF-1 and IGFBP-3. pAKT levels were used to assess IGF signaling. (**B**-**C**) Myoblasts were seeded at density of 20,000 cells/well and were grown with or without 10 ng/mL IGF-1 or 10 ng/mL IGF-1 plus 5 μg/mL IGFBP-3 in growth medium for 96 h (**B**) and cells were counted (**C**). (**D**-**E**) C2C12 myotubes were differentiated with or without 10 ng/mL IGF-1 or 10 ng/mL IGF-1 plus 5 μg/mL IGFBP-3 in differentiation medium for 96 h (**D**) and differentiation rate was measured (**E**). (**F**-**H**) C2C12 myotubes were differentiated for 96 h and treated with or without 10 ng/mL IGF-1 or 10 ng/mL IGF-1 plus 5 μg/mL IGFBP-3 in differentiation medium for 48 h (**F**) and protein degradation (**G**) and ubiquitinated protein levels were measured (**H**). Data are presented as means ± SEM. * *p* < 0.05. (TIFF 6076 kb) Additional file 8: Figure S4. PTEN loss rescues IGFBP-3-associated inhibition of C2C12 myotube differentiation. C2C12 myotubes were differentiated with or without 5 μg/mL IGFBP-3 for 96 h and myonuclei number (**A**) and muscle regulatory factors expression (**B**) were measured then. Data are presented as means ± SEM. * *p* < 0.05. (TIFF 6076 kb) Additional file 9: Figure S5. Conditioned medium from Capan-1 cells suppresses C2C12 myogenesis and promotes C2C12 myotube protein degradation. (**A**) *IGFBP3* mRNA levels were measured in si-GFP and si-IGFBP3 Capan-1 cells. (**B**) Myoblasts were seeded at density of 20,000 cells/well and were grown with Con, CM, CM-I, or CM + Ab (10 μg/mL IGFBP-3 antibody) growth medium for 96 h and cells were counted. (**C**-**E**) Myotubes were differentiated in Con, CM, CM-I, or CM + Ab (10 μg/mL IGFBP-3 antibody) differentiation medium for 96 h and differentiation rate (**C**), myonuclei number per myotube (**D**), and muscle regulatory factors expressions (**E**) were measured. (**F**) Protein levels were measured in C2C12 myotubes that were normally differentiated for 96 h and then moved to Con, CM, CM-I, or CM + Ab differentiation medium for 48 h. Data are presented as means ± SEM. * *p* < 0.05. (TIFF 6076 kb)
