# Erratum to: Pancreatic cancer cell-derived IGFBP-3 contributes to muscle wasting

**DOI:** 10.1186/s13046-016-0342-y

**Published:** 2016-05-10

**Authors:** Xiu-yan Huang, Zi-li Huang, Ju-hong Yang, Yong-hua Xu, Jiu-Song Sun, Qi Zheng, Chunyao Wei, Wei Song, Zhou Yuan

**Affiliations:** Department of General Surgery, Shanghai Jiaotong University Affiliated Sixth People’s Hospital, Shanghai, 200233 P.R China; Department of Radiology, Xuhui Central Hospital, Shanghai, 200031 P.R China; Collaborative Innovation Center of Tianjin for Medical Epigenetics, Key Laboratory of Hormone and Development (Ministry of Health), Metabolic Disease Hospital & Tianjin Institute of Endocrinology, Tianjin Medical University, Tianjin, 300070 China; Department of Cancer Immunology and AIDS, Dana-Farber Cancer Institute, 450 Brookline Ave, Boston, MA 02215 USA; Department of Medicine, Harvard Medical School, 25 Shattuck Street, Boston, MA 02115 USA; Department of Molecular Biology, Howard Hughes Medical Institute, Massachusetts General Hospital, Boston, MA USA; Department of Genetics, Harvard Medical School, Boston, MA 02115 USA

Unfortunately, in the PDF of the original version of this article [1] there was an error. The names of two of the co-first authors were included incorrectly in the footnote and read “Xiu-yang Huang” and “Juhong Yang “instead of “Xiu-yan Huang” and “Ju-hong Yang”. The sentence should have read “Xiu-yan Huang, Zi-li Huang, and Ju-hong Yang are co-first authors”.

The names have been also updated in the original article.
