# Supplementary figures and images for: Pancreatic cancer cell-derived IGFBP-3 contributes to muscle wasting
Source: J Exp Clin Cancer Res. 2016 Mar 15;35:46. doi: 10.1186/s13046-016-0317-z (PMC4791758; doi:10.1186/s13046-016-0317-z)

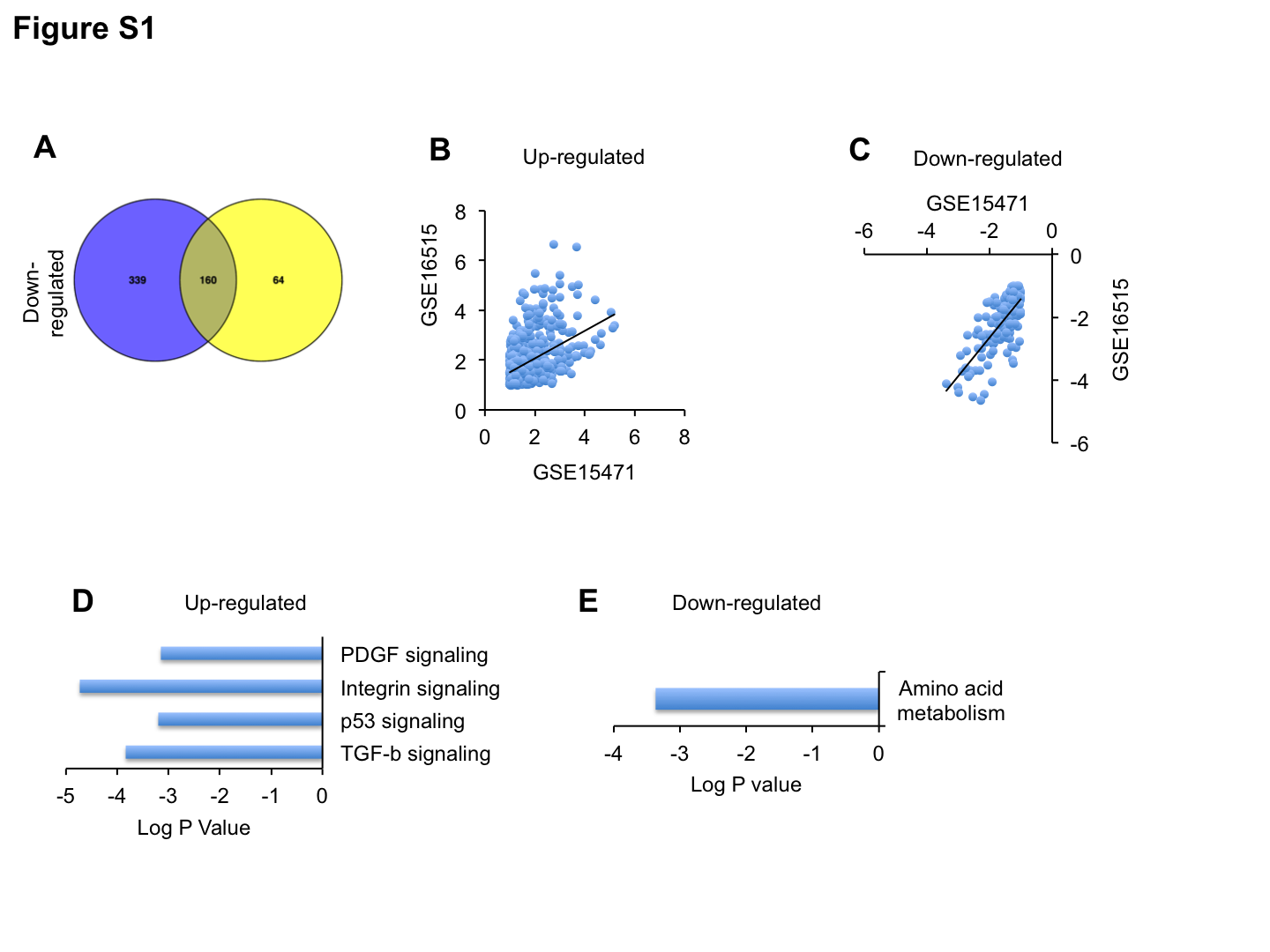

Supplement: Additional file 1: Figure S1. — Gene ontology enrichment analysis of differentially expressed genes in pancreatic tumors compared to normal tissues. (A) Significantly down-regulated genes (fold change > = 2, FDR < 0.05) were identified in two pancreatic tumor microarray datasets. The number of the overlapping genes was indicated in the middle. (B-C) Pearson’s correlation analysis of fold change (pancreatic tumor/normal tissue) of overlapping up-regulated (B) and down-regulated (C) genes in each pancreatic tumor dataset. (D-E) Significantly enriched (EASE score < 0.05) GO terms were identified from the overlapping differentially expressed genes (D, signaling pathways for up-regulated genes. E, biological process for down-regulated genes). (TIFF 6076 kb) [file 13046_2016_317_MOESM1_ESM.tiff]

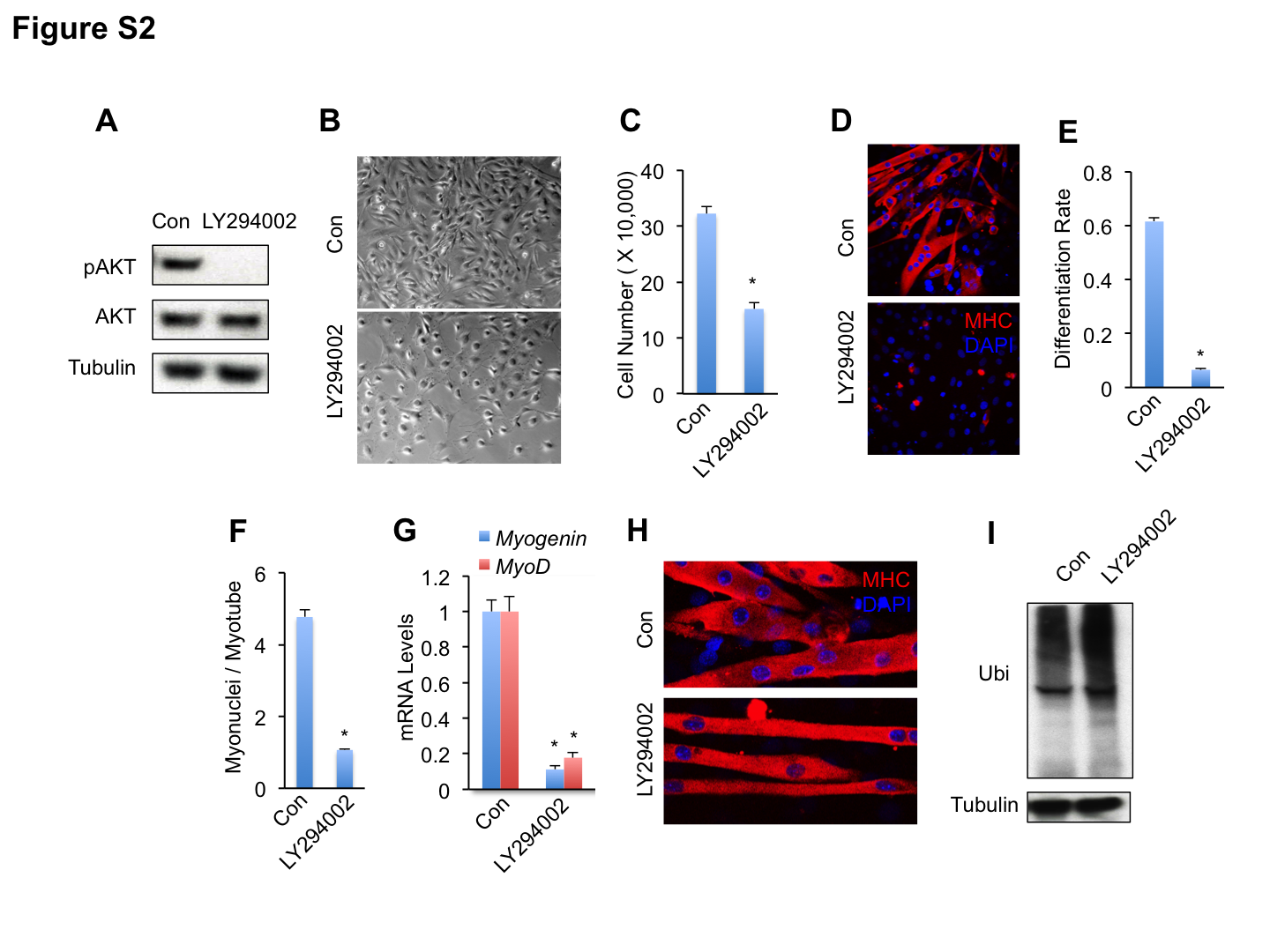

Supplement: Additional file 4: Figure S2. — Impaired IGF signaling inhibits C2C12 myogenesis and induces C2C12 myotube protein degradation. (A) IGF signaling indicated as p-AKT level in C2C12 myoblasts that were treated with or without 10 μM LY294002 for 24 h. (B-C) Myoblasts were seeded at density of 20,000 cells/well and were grown with or without 10 μM LY294002 for 96 h (B) and cells were counted (C). (D-G) C2C12 myotubes were differentiated with or without 10 μM LY294002 for 96 h (D) and differentiation rate (E), myonuclei number (F), as well as muscle regulatory factors expression (G) were measured then. (H-I) C2C12 myotubes were differentiated for 96 h and treated with 10 μM LY294002 for 48 h (H). The ubiquitinated protein levels were measured (I). Data are presented as means ± SEM. * p < 0.05. (TIFF 6076 kb) [file 13046_2016_317_MOESM4_ESM.tiff]

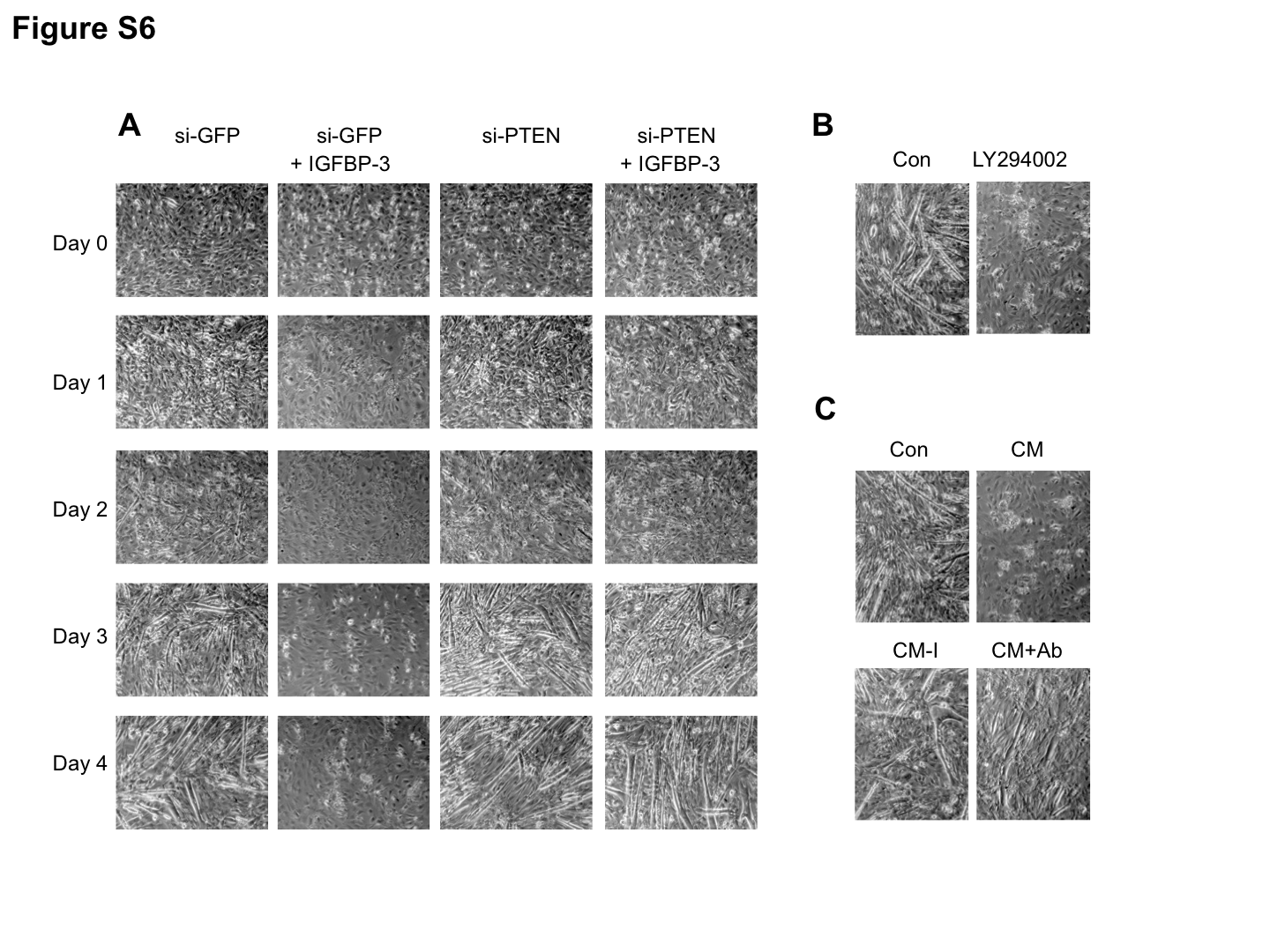

Supplement: Additional file 5: Figure S6. — IGF signaling, IGFBP-3, and conditioned medium from Capan-1 cells regulate C2C12 myotube differentiation. C2C12 myotube differentiation status. (A) si-GFP and si-PTEN myoblasts were differentiated with or without 5 μg/mL IGFBP-3 from Day 0 to Day 4. (B) Normal myoblasts were differentiated with or without 10 μM LY294002 for 96 h (Day 4). (C) Normal myoblasts were differentiated in Con, CM, CM-I, or CM + Ab (10 μg/mL IGFBP-3 antibody) differentiation medium for 96 h (Day 4). (TIFF 6076 kb) [file 13046_2016_317_MOESM5_ESM.tiff]

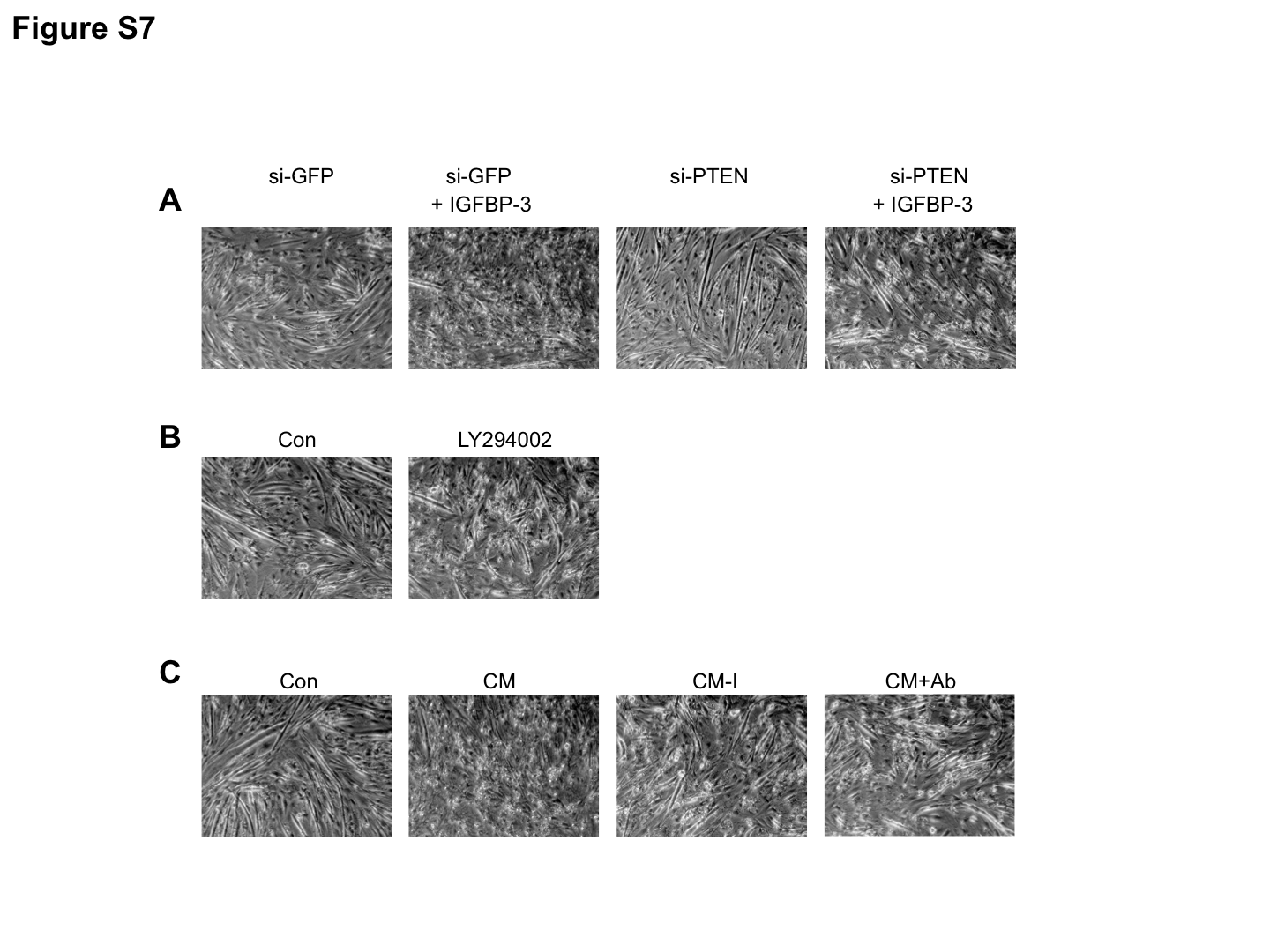

Supplement: Additional file 6: Figure S7. — IGF signaling, IGFBP-3, and conditioned medium from Capan-1 cells regulate C2C12 myotube atrophy. C2C12 myotube atrophy phenotype. (A) si-GFP and si-PTEN myoblasts were differentiated for 96 h and then treated with or without 5 μg/mL IGFBP-3 for 48 h. (B) Myotubes were differentiated for 96 h and then treated with or without 10 μM LY294002 for 48 h. (C) Myotubes were normally differentiated for 96 h and then moved to Con, CM, CM-I or CM + Ab (10 μg/mL IGFBP-3 antibody) differentiation medium for 48 h. (TIFF 6076 kb) [file 13046_2016_317_MOESM6_ESM.tiff]

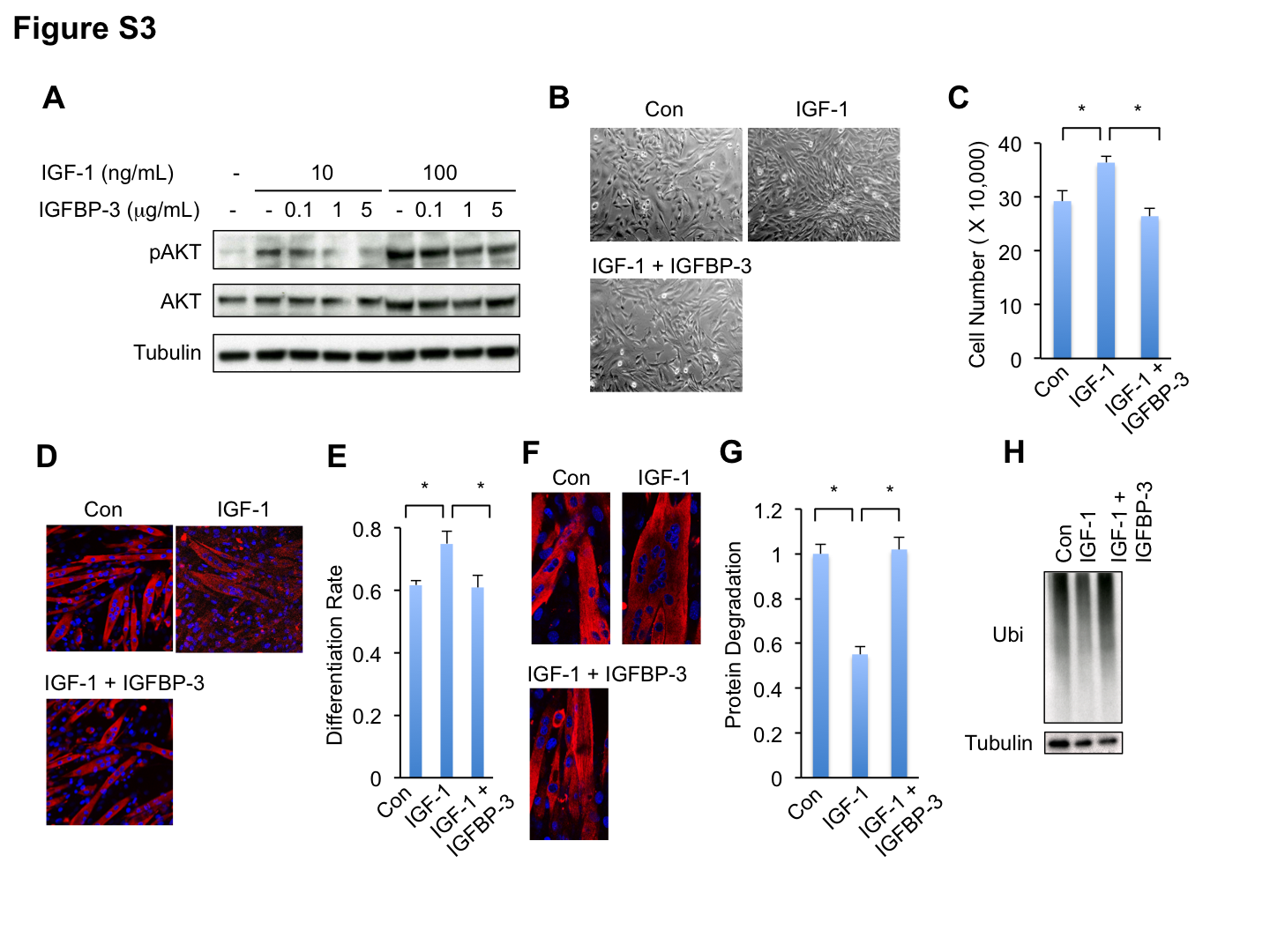

Supplement: Additional file 7: Figure S3. — IGFBP-3 counteracts IGF-1 effects on C2C12 myogenesis and C2C12 myotube protein degradation. (A) C2C12 myoblasts were deprived from serum overnight and then treated with different doses of IGF-1 and IGFBP-3. pAKT levels were used to assess IGF signaling. (B-C) Myoblasts were seeded at density of 20,000 cells/well and were grown with or without 10 ng/mL IGF-1 or 10 ng/mL IGF-1 plus 5 μg/mL IGFBP-3 in growth medium for 96 h (B) and cells were counted (C). (D-E) C2C12 myotubes were differentiated with or without 10 ng/mL IGF-1 or 10 ng/mL IGF-1 plus 5 μg/mL IGFBP-3 in differentiation medium for 96 h (D) and differentiation rate was measured (E). (F-H) C2C12 myotubes were differentiated for 96 h and treated with or without 10 ng/mL IGF-1 or 10 ng/mL IGF-1 plus 5 μg/mL IGFBP-3 in differentiation medium for 48 h (F) and protein degradation (G) and ubiquitinated protein levels were measured (H). Data are presented as means ± SEM. * p < 0.05. (TIFF 6076 kb) [file 13046_2016_317_MOESM7_ESM.tiff]

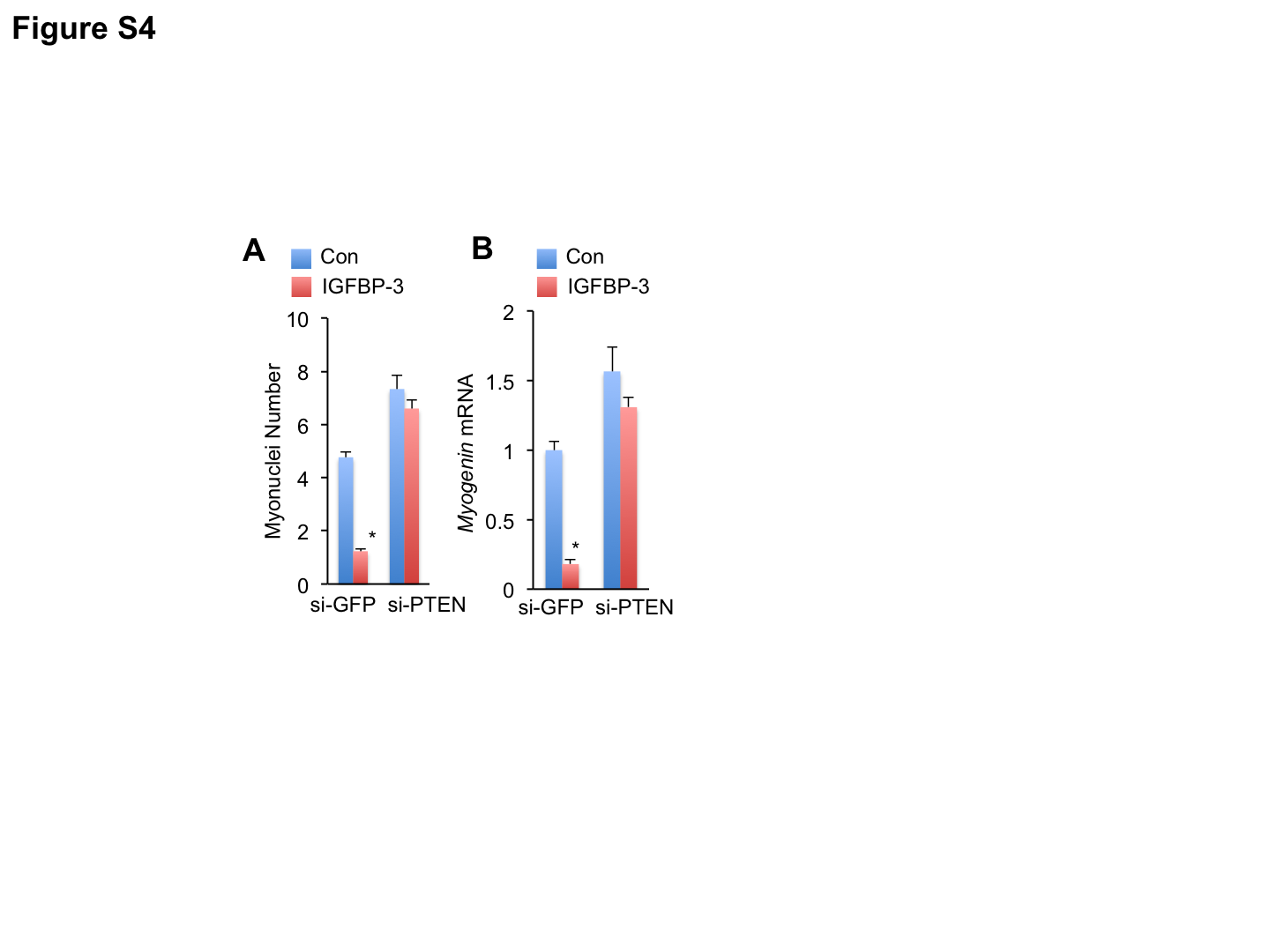

Supplement: Additional file 8: Figure S4. — PTEN loss rescues IGFBP-3-associated inhibition of C2C12 myotube differentiation. C2C12 myotubes were differentiated with or without 5 μg/mL IGFBP-3 for 96 h and myonuclei number (A) and muscle regulatory factors expression (B) were measured then. Data are presented as means ± SEM. * p < 0.05. (TIFF 6076 kb) [file 13046_2016_317_MOESM8_ESM.tiff]

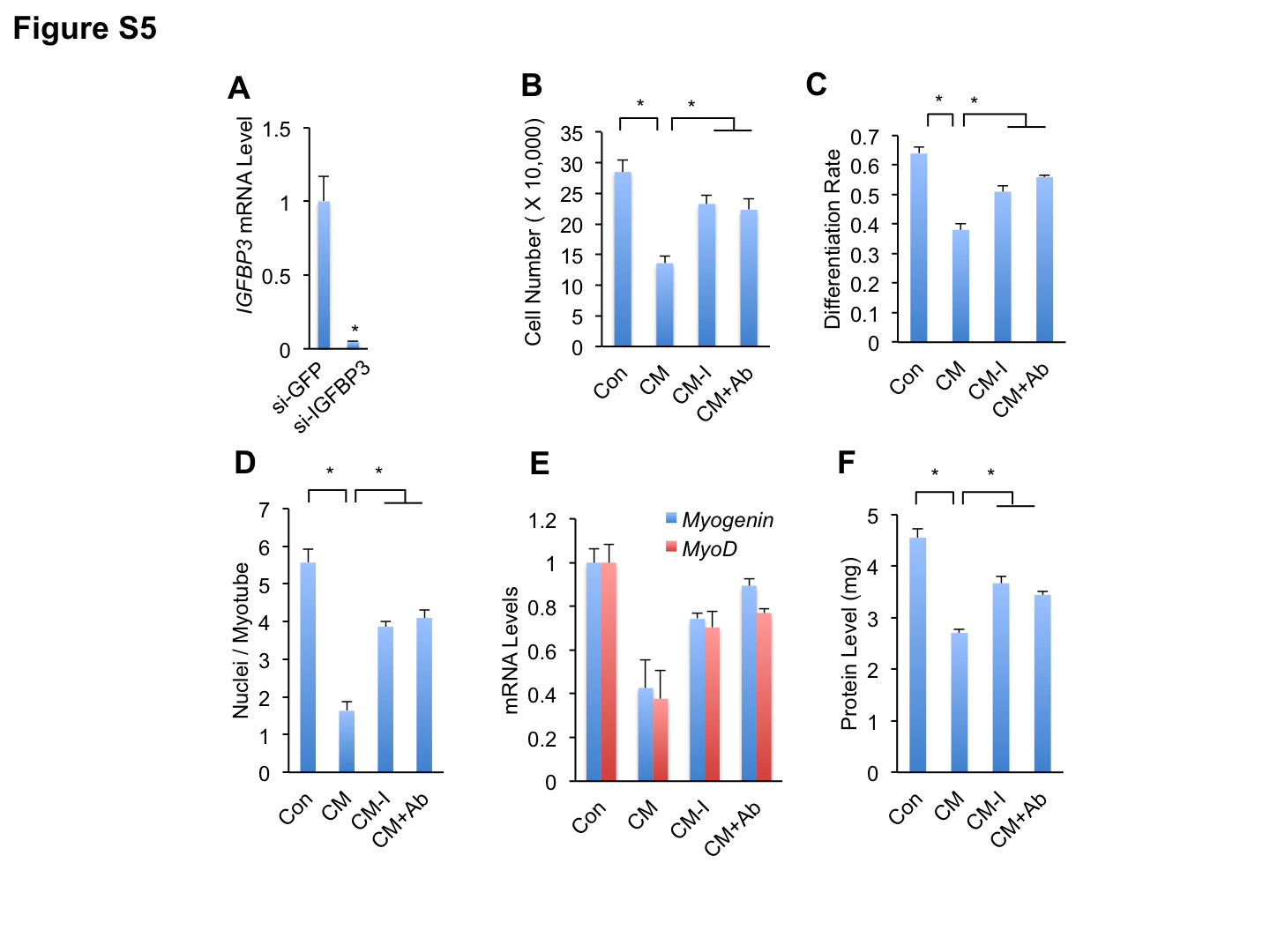

Supplement: Additional file 9: Figure S5. — Conditioned medium from Capan-1 cells suppresses C2C12 myogenesis and promotes C2C12 myotube protein degradation. (A) IGFBP3 mRNA levels were measured in si-GFP and si-IGFBP3 Capan-1 cells. (B) Myoblasts were seeded at density of 20,000 cells/well and were grown with Con, CM, CM-I, or CM + Ab (10 μg/mL IGFBP-3 antibody) growth medium for 96 h and cells were counted. (C-E) Myotubes were differentiated in Con, CM, CM-I, or CM + Ab (10 μg/mL IGFBP-3 antibody) differentiation medium for 96 h and differentiation rate (C), myonuclei number per myotube (D), and muscle regulatory factors expressions (E) were measured. (F) Protein levels were measured in C2C12 myotubes that were normally differentiated for 96 h and then moved to Con, CM, CM-I, or CM + Ab differentiation medium for 48 h. Data are presented as means ± SEM. * p < 0.05. (TIFF 6076 kb) [file 13046_2016_317_MOESM9_ESM.tiff]
